# Feeding *Aquilaria sinensis* Leaves Modulates Lipid Metabolism and Improves the Meat Quality of Goats

**DOI:** 10.3390/foods12030560

**Published:** 2023-01-27

**Authors:** Li Min, Gang Wang, Xiong Tong, Huaigu Yang, Hao Sun, Zhifei Zhang, Bin Xu, Dagang Li, Sheng Zhang, Guanghong Li

**Affiliations:** 1 Ministry of Agriculture Key Laboratory of Animal Nutrition and Feed Science in South China, Guangdong Public Laboratory of Animal Breeding and Nutrition, Guangdong Laboratory for Lingnan Modern Agriculture (Heyuan Branch), Institute of Animal Science, Guangdong Academy of Agricultural Sciences, Guangzhou 510640, China; 2 Key Laboratory of Animal Disease Prevention of Guangdong Province, Institute of Animal Health, Guangdong Academy of Agricultural Sciences, Guangzhou 510640, China; 3 Key Laboratory of Functional Foods, Ministry of Agriculture and Rural Affairs, Guangdong Key Laboratory of Agricultural Products Processing, Sericultural & Agri-Food Research Institute, Guangdong Academy of Agricultural Sciences, Guangzhou 510610, China; 4 Henan Key Laboratory of Innovation and Utilization of Grassland Resources, College of Animal Science and Technology, Henan Agricultural University, Zhengzhou 450046, China; 5 Proteomics and Metabolomics Facility, Institute of Biotechnology, Cornell University, Ithaca, NY 14853, USA; 6 Guangdong Chuangsheng Agricultural Development Co., Ltd., Shaoguan 512000, China

**Keywords:** *Aquilaria sinensis* leaves, non-conventional feed, fattening goats, lipid metabolism, meat quality

## Abstract

*Aquilaria* (*A.*) *sinensis* is a medicinal plant widely grown in tropical South China. Given the abundant pruning waste of its leaves, the use of *A. sinensis* leaves is valuable. In this study, goats were fed a diet containing 20% *A. sinensis* leaves. Compared with the basal diet, feeding *A. sinensis* leaves to goats did not affect growth performance but considerably reduced the feeding cost. Strikingly, feeding *A. sinensis* leaves resulted in a significant decrease in the blood cholesterol levels (2.11 vs. 1.49 mmol/L, *p* = 0.01) along with a significant increase in the high-density lipoprotein levels (1.42 vs. 1.82 mmol/L, *p* = 0.01). There was also a tendency to lower the content of low-density lipoprotein levels in goats (0.78 vs. 0.45 mmol/L, *p* = 0.09). Furthermore, metabolomics analysis demonstrated that the reduction in cholesterol levels occurred in both the serum (0.387-fold change) and muscle (0.382-fold change) of goats during *A. sinensis* leaf feeding. The metabolic responses to feeding *A. sinensis* leaves suggest that the activation of lipolysis metabolism might happen in goats. These observed changes would be conducive to improving animal health and meat quality, ultimately benefiting human health.

## 1. Introduction

*Aquilaria* (*A.*) *sinensis* is a highly valuable medicinal plant belonging to the genus Aquilaria in the family Thymelaeaceae; it is native to China and widely grown in tropical South China [1]. Notably, *A. sinensis* has already been demonstrated to be rich in bioactive compounds (over 249 compounds have been isolated and identified) that exhibit a wide range of biological effects [2]. The major chemical constituents of *A. sinensis* leaves identified so far are amino acids, flavonoids, phenolic acids, lipids, and xanthones [3]. In order to maximize the commercial value of the *A. sinensis* industry, apart from its medicinal value, the development of *A. sinensis* leaves has been conducted in recent years [4]. The safety evaluation of the *A. sinensis* leaves was performed for toxicology in animal and clinical trials [5], and both led to guaranteed safety. Because of the considerable production and pruning waste, efforts are increasingly being made to maximize the use of the prodigal leaves. The utilization of *A. sinensis* leaves as feedstock might offer insight into new applications and benefit the relevant field.

In the last decade, the sources of feedstuff have become increasingly more expensive and difficult to access. Thus, there is a growing number of experimental investigations in promising non-conventional feed resources for improving goat production, such as *pterocarpus lucens* leaves [6], cassava leaves [7], *vachellia karroo* leaves [8], barberry leaves [9], *moringa* leaves [10], orange leaves [11], *larrea divaricata* and *acacia aroma* leaves [12], fenugreek leaves [13], paulownia leaves [14], etc. In general, farmers feed goats with relatively low-quality straw and crop residues, which might not satisfy adequate levels of performance. Partially substituting these ingredients with leaves will reduce the feeding cost and improve production performance. In this study, we first evaluated and confirmed that goats like to ingest *A. sinensis* leaves (Figure S1) and then analyzed the leaves’ nutritional value. We found *A. sinensis* leaves to be a promising non-conventional feed resource for goat production. We also identified the specific bioactive compounds from *A. sinensis* leaves using UPLC-MS/MS analysis to illustrate their potential function in goats.

Therefore, the objective of this experiment was to investigate the effects of feeding *A. sinensis* leaves on production performance and the corresponding metabolite profile changes in the serum and muscles of goats.

## 2. Materials and Methods

### 2.1. Animals and Experimental Design

All animal procedures performed in this study were approved and supervised by the Animal Care and Use Committee of the Guangdong Academy of Agricultural Sciences (IAS2020001, Guangzhou, China).

Twelve three-month-old healthy, castrated male Chinese native growing goat kids with the same genetic background and similar weight (16.7 ± 1.6 kg) were randomly assigned to two different total mixed ration (TMR) diets: (1) the control group (CON, *n* = 6) with a normal diet, and (2) the *A. sinensis* leaves feeding group (ASL, *n* = 6) with a diet containing 20% *A. sinensis* leaves (dry matter basis). The diets and the dosage of *A. sinensis* leaves were formulated based on the nutrient requirements of goats in this phase (Table 1). The experiment lasted three months.

Animals in both groups were housed in individual units with ad libitum access to diets and water. At the end of the experiment, the goats were weighed after fasting for 12 h with free access to water. Serum samples were obtained via jugular venipuncture and centrifuged at 3000× *g* for 10 min (4 °C). Subsequently, all goats were slaughtered according to the related provisions of animal welfare. The longissimus muscle from the lumbar region was collected from the right side of each carcass and culled out of the connective and fat tissues. Muscle samples were cut into 2 cm^3^ cubes. All samples were frozen in liquid nitrogen and stored at −80 °C until analyzed.

### 2.2. Nutritional Ingredients Analysis of A. sinensis Leaves

The fresh *A. sinensis* leaves were dried in a forced-draft oven at 65 °C for 48 h. The dry matter was calculated by the weight change before and after the drying. The dried samples were ground with a cutting mill and then passed through a 1 mm sieve for further analysis. Organic matter, crude protein, and ether extract were determined using Association of Official Analytical Chemists (AOAC) methods. Neutral detergent fiber and acid detergent fiber were analyzed using an Ankom fiber analyzer according to the previous study [15].

### 2.3. Bioactive Compounds Analysis of A. sinensis Leaves

The freeze-dried *A. sinensis* leaves were crushed using a mixer mill with a zirconia bead for 1.5 min at 30 Hz. The powder was weighted at 100 mg and extracted with 0.6 mL of 70% aqueous methanol at 4 °C overnight. The extracts were centrifugated at 10,000× *g* for 10 min, and then they were absorbed and filtrated (0.22 μm pore size, ANPEL, Shanghai, China). The sample extracts were analyzed using a UPLC-MS/MS system (UPLC, Shim-pack UFLC SHIMADZU CBM30A system coupled with a triple quadrupole-linear ion trap mass spectrometer, Applied Biosystems 4500 Q TRAP). Chromatographic separation was performed on a Waters ACQUITY UPLC HSS T3 C18 column (1.8 µm, 2.1 mm × 100 mm). The mobile phase consisted of (A) pure water with 0.04% acetic acid and (B) the solvent acetonitrile with 0.04% acetic acid. The gradient program started with 5% B at 350 µL/min, followed by a 10 min ramping to 95% B, a 1 min hold at 95% B, and a quick switch to 5% B within 0.1 min. The column was re-equilibrated with 5% B for 2.9 min prior to the next run. 

The detection of metabolites was performed by MetWare Biotechnology Co., Ltd. (Wuhan, China). Metabolites were distinguished by comparing the m/z values of precursor ions, retention times, and fragmentation patterns with the standards in a database compiled by MetWare Biotechnology Co., Ltd., and quantitative detection was performed according to a multiple reaction monitoring pattern [16,17].

The total flavonoid, isoflavone, and anthocyanin contents in *A. sinensis* leaves were detected by spectrophotometry. The composition of fatty acids in *A. sinensis* leaves was analyzed by a trace GC ultra gas chromatograph. The contents of hydrolyzed amino acids in *A. sinensis* leaves were determined by the amino acid analyzer. 

### 2.4. Serum Biochemistry Analysis of Goats

The concentrations of total protein, albumin, glutamic pyruvic transaminase, glutamic oxalacetic transaminase, glucose, blood urea nitrogen, uric acid, total cholesterol, triglyceride, high-density lipoprotein, and low-density lipoprotein in serum were analyzed and determined by a semiautomatic biochemistry analyzer using commercial kits (Nanjing Jiancheng Biological Engineering Institute, Nanjing, China).

### 2.5. UPLC-MS/MS Metabolomics Analysis of Serum and Muscle Samples

Serum and muscle samples were thawed on ice. To each 50 μL serum sample, 150 μL of ice-cold methanol was added; the mixture was whirled for 3 min, and then centrifuged with 10,000× *g* at 4 °C for 15 min. Each muscle sample containing 50 mg of tissue was homogenized in 1 mL of ice-cold methanol/water (70% *v*/*v*) using a superfine homogenizer (30 Hz for 3 min). After whirling the mixture for 1 min, samples were centrifuged with 10,000× *g* at 4 °C for 10 min. The supernatant collected from each serum and muscle sample was used for UPLC-MS/MS analysis as described above.

### 2.6. Statistical Analysis

Data for growth performance, serum biochemical indexes, and quantitatively determined cholesterol contents by HPLC were analyzed using the GLM procedure of SAS (SAS Institute Inc., Cary, NC, USA). The Student’s t test was used for the comparison of sample data. Standard errors of the mean (SEM) were represented. Differences were considered significant at *p* < 0.05, and significantly different trends were defined at 0.05 < *p* < 0.1.

For metabolomics analysis, the relative relevance of each metabolite to the orthogonal partial least squares discriminant analysis (OPLS-DA) model was determined. The variable importance in projection (VIP) parameters were extracted from the OPLS-DA results. VIP > 1 and fold change > 2 or <0.5 were used for determining the significantly different metabolites.

## 3. Results

### 3.1. Nutritional Ingredients and Bioactive Compounds of A. sinensis Leaves

As shown in Table 2, *A. sinensis* leaves could be utilized as a potential source of feedstock for ruminants.

**Figure 1:**
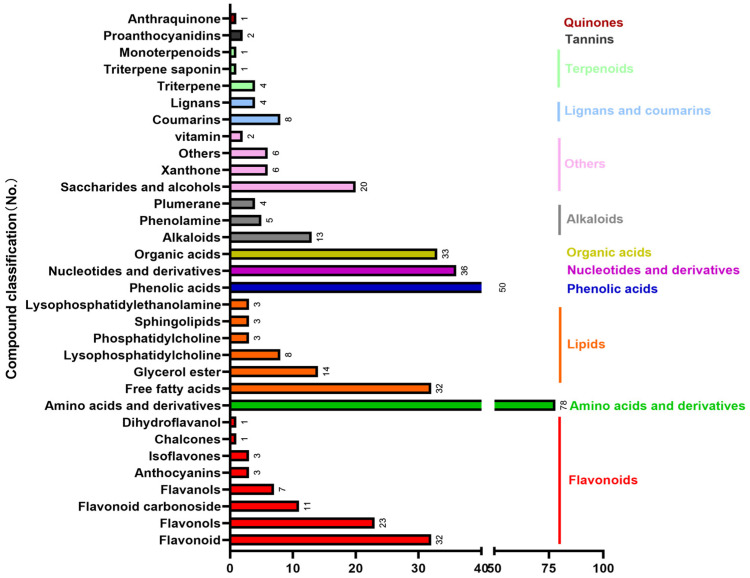
The classification of 418 bioactive compounds in *A. sinensis* leaves identified in this study.

**Figure 2:**
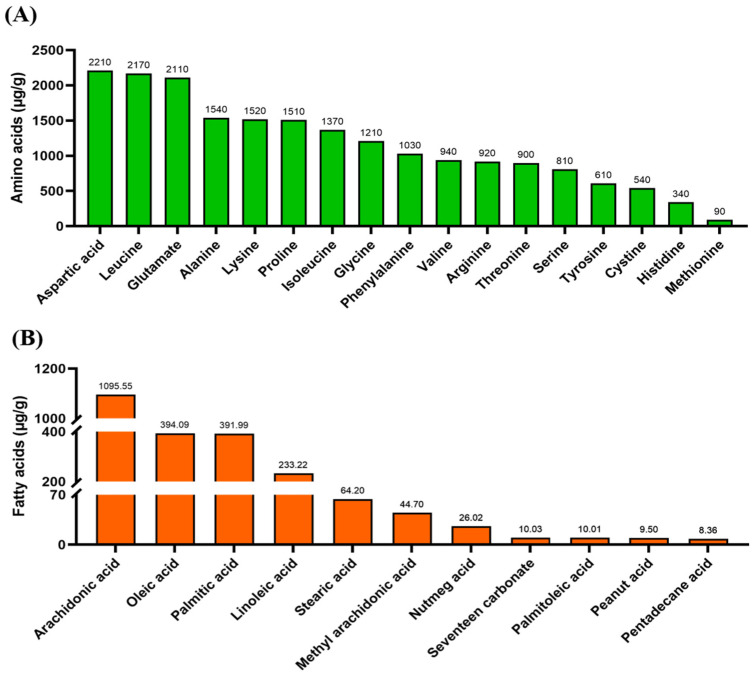
The contents of amino acids and fatty acids in *A. sinensis* leaves obtained by the amino acid analyzer and GC ultra gas chromatograph, respectively. (**A**) The contents of amino acids in *A. sinensis* leaves. (**B**) The contents of fatty acids in *A. sinensis* leaves.

UPLC-MS/MS analysis identified a total of 418 bioactive compounds from *A. sinensis* leaves (Table S1). Of these, we identified 81 flavonoids, 78 amino acids and derivatives, 63 lipids, 50 phenolic acids, and other bioactive compounds (Figure 1). *A. sinensis* leaves are particularly rich in flavonoids; the contents of total flavonoids, isoflavones, and anthocyanin were 8.14 mg/g, 4.02 mg/g, and 188.32 nmol/g, respectively. The contents of 17 amino acids and fatty acids in *A. sinensis* leaves are presented in Figure 2. Results indicated that *A. sinensis* leaves contained high levels of unsaturated fatty acids.

### 3.2. Effects of Feeding A. sinensis Leaves on Growth Performance and Serum Biochemical Indexes of Goats

Compared with the control group, no effect was observed on the growth performance of goats after feeding *A. sinensis* leaves. There were no significant differences between the two groups on the indexes of initial weight, final weight, average daily intake, and average daily gain (Table 3).

Additionally, no significant differences were observed in the serum concentrations of total protein, albumin, glutamic pyruvic transaminase, glutamic oxyacetic transaminase, glucose, blood urea nitrogen, and triglyceride between the two groups (Table 3). Intriguingly, we found that feeding *A. sinensis* leaves significantly decreased blood cholesterol levels while significantly increasing high-density lipoprotein levels in goats (*p* < 0.05, Table 3). Meanwhile, there was a consistent decreasing tendency in the content of uric acid and low-density lipoprotein in goats’ blood after feeding *A. sinensis* leaves (*p* < 0.1, Table 3).

### 3.3. Changes of the Metabolites and the Corresponding Pathways of Serum and Muscle in Goats

**Figure 3:**
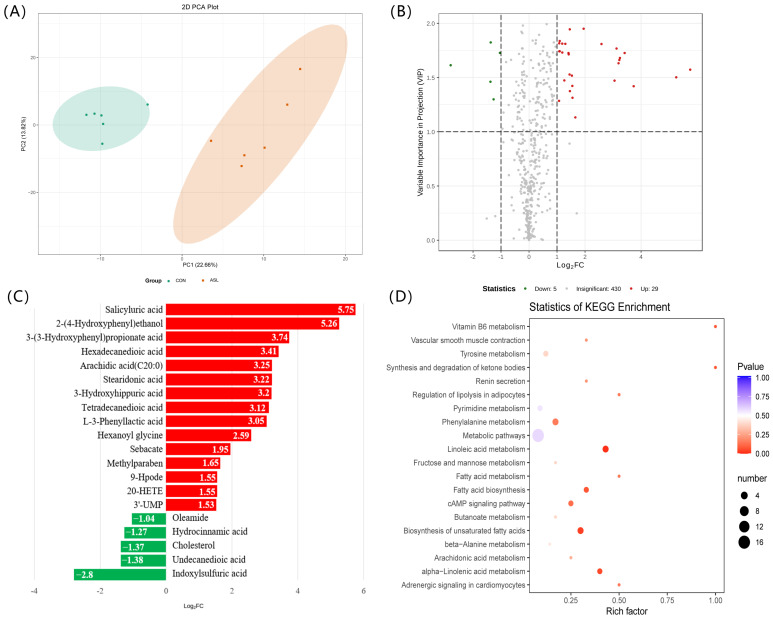
Serum metabolomics analysis reveals changes in the metabolites and their associated signal pathways in response to feeding *A. sinensis* leaves to goats. (**A**) The PCA score plot of serum metabolome distribution shows clear separate clusters for goats with different diets. (**B**) A volcano plot showing differential serum metabolites between the CON and ASL groups. (**C**) Top 20 differential serum metabolites between the CON and ASL groups. (**D**) The KEGG enrichment analysis suggests significant changes occurred in the signal pathways of unsaturated fatty acid biosynthesis, linoleic acid metabolism, and α -linolenic acid metabolism. CON: a normal diet; ASL: a diet containing 20% *A. sinensis* leaves. Red indicates an increase, while green indicates a decrease. 20-HETE: 20-hydroxy-5Z,8Z,11Z,14Z-eicosatetraenoic acid.

**Figure 4:**
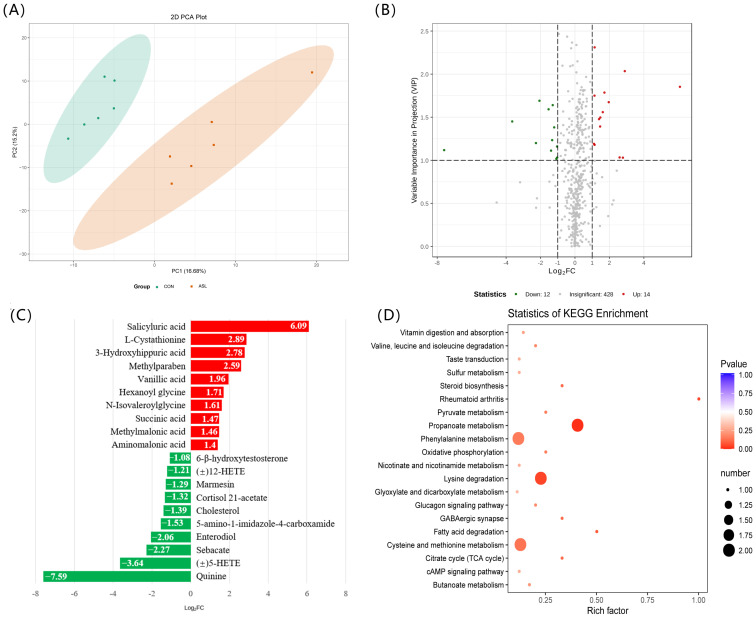
Muscle metabolomics analysis reveals changes in the metabolites and the signal pathways during feeding *A. sinensis* leaves to goats. (**A**) PCA scores a plot of muscle metabolome distribution according to the diet of goats. (**B**) A volcano plot showing differential muscle metabolites between CON and ASL groups. (**C**) Top 20 differential muscle metabolites between the CON and ASL groups. (**D**) The KEGG enrichment analysis suggests significant changes occurred in the signal pathways of phenylalanine metabolism, lysine degradation, and cysteine and methionine metabolism. CON: a normal diet, ASL: a diet containing 20% *A. sinensis* leaves. Red indicates an increase, while green indicates a decrease. (±)12-HETE: (±)12-hydroxy-5Z,8Z,10E,14Z-eicosatetraenoic acid, 5-amino-1-imidazole-4-carboxamide: 5-amino-1-[3,4-dihydroxy-5-(hydroxymethyl)oxolan-2-yl]imidazole-4-carboxamide, (±)5-HETE: (±)5-hydroxy-6E,8Z,11Z,14Z-eicosatetraenoic acid.

To further elucidate the metabolic responses of feeding *A. sinensis* leaves to goats, serum and muscle metabolomics data were obtained in parallel (Tables S2 and S3). The results of the principal component analysis (PCA) indicated obvious discrimination between the two groups (Figure 3A and Figure 4A). The supported information of PCA scores plot and an orthogonal partial least squares-discriminant analysis (OPLS-DA) score plot of serum and muscle metabolome distribution according to the diet of goats were also presented in Tables S4 and S5 and Figure S2.

After feeding *A. sinensis* leaves, 29 serum metabolites (also including cholesterol) were significantly increased, while five metabolites declined (Figure 3B and Table 4). Therein, the contents of unsaturated fatty acids, such as α-linolenic acid and γ-linolenic acid, were found to be higher in serum from goats fed *A. sinensis* leaves. The details of the top 20 differential metabolites between the two groups are shown in Figure 3C, based on linear discriminant analysis and effect size analysis. The KEGG enrichment analysis of differential metabolites revealed that significant changes occurred in the key metabolic pathways involving unsaturated fatty acid biosynthesis, linoleic acid metabolism, and α-linolenic acid metabolism (Figure 3D).

Meanwhile, a total of 26 differential compounds in the longissimus muscle of goats in response to feeding of *A. sinensis* leaves, have been identified using metabolomics analysis. Of these, 14 metabolites were significantly increased, while 12 metabolites were significantly decreased in the *A. sinensis* leaves feeding group (Figure 4B and Table 5). In this process, it is worth noting that the contents of cholesterol in muscle were reduced, and the contents of flavor substances in muscle, such as succinic acid and γ-glutamyl-leucine, were increased. The top 20 differential metabolites in muscle are displayed in Figure 4C. The KEGG enrichment analysis indicated that the differential metabolites in muscle were associated with phenylalanine metabolism, lysine degradation, cysteine, and methionine metabolism pathways (Figure 4D).

## 4. Discussion

Previous studies have reported *A. sinensis* leaves’ analgesic, antiarthritic, antidiabetic, anti-inflammatory, antimicrobial, antioxidant, and hepatoprotective roles [18,19]. Given the abundance of bioactive compounds in *A. sinensis* leaves, there could potentially be several pharmacological effects for goats. The analysis of serum biochemical indexes after feeding *A. sinensis* leaves points to marked differences in the metabolic state by providing useful insights on the evaluation of metabolites associated with body fluid distribution in goats [20]. In the present study, we found that feeding *A. sinensis* leaves might most likely to play a role in hypolipidemic in goats, as indicated by decreased cholesterol and low-density lipoprotein and increased high-density lipoprotein. Moreover, the decrease in cholesterol has also been verified by serum metabolomics analysis. Alam et al. [18] demonstrated that administering 200 and 400 mg/kg of an extract of *A. sinensis* leaves would significantly reduce the cholesterol levels in rats. Consistent with this study, goats treated with the extract of *A. sinensis* plants exhibited significantly increased activity of the liver X receptor in HepG2 cells [21]. It is well known that the liver X receptor could correct sterol overload by promoting cholesterol disposal from the cell [22]. Furthermore, HepG2 cells treated with the extract of *A. sinensis* plants also resulted in a significant increase in PPARα [21], which is known for its hypolipidemic effect. As shown in Table S1, *A. sinensis* leaves are rich in flavonoids and phenolic acids (especially genkwanin, kaempferol, and mangiferin), which have already been reported to bind well with PPARs or act as PPAR agonists [23,24]. On the basis of a previous experiment with rats and cells and this study (bioactive compounds of *A. sinensis* leaves and serum biochemical indexes of goats), we suspect that the *A. sinensis* leaves’ hypolipidemic role in goats occurs through the modulation of a lipid metabolism signaling pathway that reduces cholesterol and low-density lipoprotein and enhances high-density lipoprotein. This inference merits further research and verification at the cellular level.

The compounds present in *A. sinensis* leaves and shown as serum differential metabolites in goats include 3-hydroxybutyrate, α-linolenic acid, γ-linolenic acid, palmitoleic acid, and N-acetylneuraminic acid (Table S1 and Table 4). As shown in the present study, *A. sinensis* leaves contained high levels of unsaturated fatty acids (Table 4). Ahmed et al. [25] demonstrated that a diet rich in linolenic acids would decrease the cholesterol contents in the blood and muscles of goats, while linolenic acids would induce the upregulation of PPARα and PPARγ expression [26,27]. Integrated with the above research findings, Liang et al. [28] and Liu et al. [29] concluded that linolenic acids could reduce lipid accumulation and blood lipid, as indicated by the decrease in cholesterol, low-density lipoprotein, and adipose tissue weight and the increase in the gene and protein expressions of lipid catabolism. In the current study, our data support the notion that supplementation of *A. sinensis* leaves (enriched with linolenic acids, flavonoids, and phenolic acids) promoted lipolysis in goats and ultimately reduced the contents of cholesterol in muscle.

It is curious that the increase in methylparaben concentration was observed in both serum and muscle tissue of goats fed with *A. sinensis* leaves (Figure 3C and Figure 4C). As we know, methylparaben is a conjugate between 4-hydoxybenzoic acid and methanol synthesized by two enzymatic reactions [30]. We speculated that the methylparaben was derived from 4-hydroxybenzoic acid in the leaves of *A. sinensis* (Table S1). This biosynthetic process might occur in the rumen.

Methylmalonic acid and succinic acid appeared in the list of the muscle differential metabolites in goats and were also derived from *A. sinensis* leaves (Table S1 and Table 5). Succinic acid, a natural flavor enhancer, has been reported to confer an umami taste for meat [31] and plays a significant role in determining meat color stability [32]. Additionally, succinic acid can induce muscle fiber remodeling [33] and positively correlate with meat shear force (toughness) [34]. We observed that feeding *A. sinensis* leaves can give rise to an increasing level of succinic acid in the muscle of goats, suggesting the enhancement of the flavor and texture of the meat. To obtain a comprehensive insight into the flavor and texture, a sensory evaluation should be conducted by the potential consumers of this product in the future.

According to our findings, the amount of cholesterol in muscle was reduced during this process. Consumption of dietary cholesterol is a potential cause of human disease, including atherosclerosis and coronary heart disease. The World Health Organization advised that dietary cholesterol intake should not exceed 300 mg daily [35]. It is generally known that the sources of dietary cholesterol are primarily derived from foods of animal origin, such as meat, eggs, fish roe, and animal viscera [36]. In order to validate the decreasing level of cholesterol in meat, we further quantitatively determined the cholesterol contents by HPLC, as referred to in the previous study [37]. The results confirmed that feeding *A. sinensis* leaves dramatically lowered cholesterol levels in the longissimus muscle (CON: 889.02 mg/kg vs. ASL: 366.28 mg/kg, *p* < 0.01), thus improving the meat quality of goats. In consequence, we suspect that feeding *A. sinensis* leaves to goats could open an effective alternative strategy for humans to reduce cholesterol intake from mutton and benefit their health.

## 5. Conclusions

Overall, from the analysis of nutritional ingredients and the feeding experiment, it can be concluded that *A. sinensis* leaves can be used as a non-conventional feedstuff for goat production. Despite feeding *A. sinensis* leaves to goats, it did not improve their growth performance but could considerably reduce the feeding cost.

**Figure 5:**
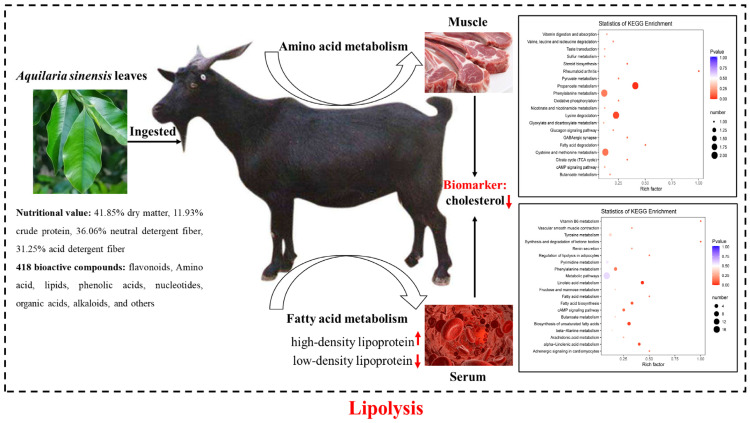
A schematic diagram of responses to feeding *A. sinensis* leaves to goats. *A. sinensis* leaves are rich in linolenic acids, flavonoids, and phenolic acids that play the role of hypolipidemic in goats via the modulation of the lipid metabolism signaling pathway. The reduction in blood cholesterol and low-density lipoprotein levels and the increase in high-density lipoprotein levels in goats in response to the diet strongly implied the activation of lipolysis metabolism during feeding *A. sinensis* leaves.

Furthermore, this study demonstrated that *A. sinensis* leaves contained a broad array of 418 bioactive compounds, mainly belonging to flavonoids, amino acids, lipids, and phenolic acids. The diet effects of feeding *A. sinensis* leaves to goats are proposed and summarized in Figure 5. When *A. sinensis* leaves are ingested via the diet of goats, the most important responsive metabolic pathways in serum are focused on fatty acid metabolism, while those in muscle are reflected in amino acid metabolism. Further research is warranted to illustrate the different metabolic pathways that occur in the different parts of goats. *A. sinensis* leaves are rich in linolenic acids, flavonoids, and phenolic acids that play the role of hypolipidemic in goats via the modulation of the lipid metabolism signaling pathway. This inference needs further research and verification. The reduction in blood cholesterol and low-density lipoprotein levels and an increase in high-density lipoprotein levels in goats in response to the diet strongly implied the activation of lipolysis metabolism during feeding with *A. sinensis* leaves. One of the noteworthy biomarkers found in this study is cholesterol, which changes consistently based not only on serum biochemical analysis and quantitative determination in the muscle but also on untargeted metabolomics analysis of serum and muscle tissue.

The decrease in cholesterol content in serum and muscle suggested that the diet of *A. sinensis* leaves benefits goat health and improves goat meat quality for the benefit of human health.

## Figures and Tables

**Table 1:** Dietary ingredients and nutrient composition (dry matter basis) of fattening goats between two groups.

Item	Diets	
	CON	ASL
**Ingredients (%)**		
Corn straw silage	48.8	33.8
*A. sinensis* leaves		20.0
Peanut vine	15.5	10.5
Corn	15.2	15.5
Soybean meal	13.8	13.5
Wheat bran	4.1	4.1
Salt	0.5	0.5
Dicalcium phosphate	1.1	1.1
Limestone	0.5	0.5
Premix ^1^	0.5	0.5
**Nutrient composition (%)**		
Crude protein	10.98	11.05
Neutral detergent fiber	41.15	38.50
Acid detergent fiber	25.64	24.82
Calcium	0.64	0.64
Phosphorus	0.41	0.41
Digestible energy (MJ/kg)	10.76	10.35

^1^ Premix contained the following per kg of diet: vitamin A 1800 IU, vitamin D 2750 IU, vitamin E 1480 IU, iron 32 mg, zinc 22 mg, manganese 14 mg, copper 2.4 mg, iodine 0.5 mg, selenium 0.2 mg, and cobalt 0.08 mg.

**Table 2:** Nutrient composition (dry matter basis) of *A. sinensis* leaves.

Item	Contents (%)
Dry matter	41.85
Organic matter	37.10
Crude protein	11.93
Crude fat	3.46
Neutral detergent fiber	36.06
Acid detergent fiber	31.25

**Table 3:** Effects of feeding *A. sinensis* leaves on the growth performance and serum biochemical indexes of goats.

Item	Diets		SEM	*p* Value
	CON	ASL		
**Growth performance**				
Initial weight (kg)	16.4	17.0	1.13	0.32
Final weight (kg)	21.3	21.9	1.33	0.45
Average daily intake (g/d)	697.8	705.4	60.8	0.78
Average daily gain (g/d)	53.5	53.7	6.51	0.92
**Serum biochemical indexes**				
Total protein (g/L)	68.9	61.4	3.39	0.92
Albumin (g/L)	24.4	24.8	1.08	0.14
Glutamic pyruvic transaminase (U/L)	11.2	13.6	0.78	0.51
Glutamic oxyacetic transaminase (U/L)	64.5	83.9	9.87	0.57
Glucose (mmol/L)	5.85	3.62	0.17	0.23
Blood urea nitrogen (mmol/L)	8.68	8.78	0.79	0.48
Uric acid (µmol/L)	25.2	16.3	1.25	0.06
Cholesterol (mmol/L)	2.11	1.49	0.12	0.01
Triglyceride (mmol/L)	0.34	0.39	0.07	0.40
High-density lipoprotein (mmol/L)	1.42	1.82	0.11	0.01
Low-density lipoprotein (mmol/L)	0.78	0.45	0.04	0.09

**Table 4:** Serum metabolites that differ significantly in the goat samples after feeding *A. sinensis* leaves using UPLC-MS/MS analysis.

Compounds	Classification	VIP	Fold Change	Log2FC
Indoxylsulfuric acid	Organic acid and its derivatives	1.612864	0.143783	−2.798038
Hydrocinnamic acid	Organic acid and its derivatives	1.298589	0.41591	−1.265657
Undecanedioic acid	Fatty acyls	1.460975	0.384958	−1.377228
Cholesterol	Lipids	1.823414	0.387121	−1.369145
Oleamide	Lipids fatty acids	1.727046	0.486523	−1.039421
Salicyluric acid	Benzene and substituted derivatives	1.572335	53.98397	5.754459
2-(4-Hydroxyphenyl)ethanol	Benzene and substituted derivatives	1.501871	38.21139	5.255931
4,4′-Methylenedianiline	Benzene and substituted derivatives	1.374475	2.748404	1.458594
3-(3-Hydroxyphenyl)Propionate acid	Organic acid and its derivatives	1.41958	13.32459	3.736019
3-Hydroxyhippuric acid	Organic acid and its derivatives	1.631329	9.162949	3.195812
L-3-Phenyllactic acid	Organic acid and its derivatives	1.471008	8.295528	3.052334
Sebacate	Organic acid and its derivatives	1.949887	3.862228	1.949434
N-Oleoyl glycine	Organic acid and its derivatives	1.944144	2.741058	1.454733
Imidazoleacetic acid	Organic acid and its derivatives	1.527683	2.726868	1.447245
Malonicacid	Organic acid and its derivatives	1.741892	2.135244	1.094401
3-Hydroxybutyrate	Organic acid and its derivatives	1.741623	2.115696	1.081132
Hexadecanedioic acid	Lipids fatty acids	1.72588	10.64918	3.412671
Arachidic Acid(C20:0)	Lipids fatty acids	1.680261	9.482547	3.245275
Stearidonic acid	Lipids fatty acids	1.661258	9.344554	3.224126
Tetradecanedioic acid	Lipids fatty acids	1.767233	8.718257	3.12404
α-Linolenic acid(C18:3N3)	Lipids fatty acids	1.713567	2.66958	1.416613
γ-Linolenic acid(C18:3N6)	Lipids fatty acids	1.725919	2.653937	1.408134
Palmitoleic acid(C16:1)	Lipids fatty acids	1.810366	2.455625	1.29609
8,15-Dihete	Lipids fatty acids	1.730287	2.278096	1.187828
Hexanoyl glycine	Amino acid metabolomics	1.808671	6.006694	2.586571
N-Acetylneuraminic acid	Amino acid metabolomics	1.473051	2.395256	1.26018
Methylparaben	Benzoic acid and its derivatives	1.132065	3.146752	1.653863
9-Hpode	Lipids	1.313142	2.93127	1.551526
20-HETE [20-hydroxy-5Z,8Z,11Z,14Z-eicosatetraenoic acid]	Oxidized lipid	1.422309	2.920729	1.546329
9,10-DiHOME [(±)9,10-dihydroxy-12Z-octadecenoic acid]	Oxidized lipid	1.28463	2.101768	1.071603
3′-UMP	Nucleotide metabolomics	1.517275	2.888509	1.530325
4-Pyridoxic acid	Pyridine and pyridine derivatives	1.812439	2.275612	1.186255
Epinephrine	Hormones	1.836576	2.13752	1.095938
L-Rhamnose	Carbohydrate metabolomics	1.815123	2.111195	1.07806

**Table 5:** The longissimus muscle metabolites that differ significantly in the goat samples after feeding *A. sinensis* leaves using UPLC-MS/MS analysis.

Compounds	Classification	VIP	Fold Change	Log2FC
Quinine	Alkaloid	1.117945	0.005174	−7.59449
(±)5-HETE [(±)5-hydroxy−6E,8Z,11Z,14Z-eicosatetraenoic acid]	Oxidized lipid	1.45057	0.08001	−3.643675
Sebacate	Organic acid and its derivatives	1.199688	0.207823	−2.266575
Enterodiol	Phenols and its derivatives	1.690837	0.239295	−2.063138
5-amino-1-[3,4-dihydroxy-5-(hydroxymethyl)oxolan-2-yl]imidazole-4-carboxamide	Nucleotide metabolomics	1.591578	0.345404	−1.533644
Cholesterol	Lipids	1.112062	0.381854	−1.388905
Cortisol 21-acetate	Lipids	1.233468	0.39921	−1.324779
12-Hete	Lipids	1.159916	0.485659	−1.041984
Marmesin	Carbohydrate metabolomics	1.639964	0.408265	−1.292422
(±)12-HETE [(±)12-hydroxy-5Z,8Z,10E,14Z-eicosatetraenoic acid]	Oxidized lipid	1.381799	0.433116	−1.207174
6-β-hydroxytestosterone	Hormones	1.017614	0.47224	−1.082408
3-Indolebutyric acid	Indole and its derivatives	1.031324	0.488706	−1.032961
Salicyluric acid	Benzene and substituted derivatives	1.852642	68.24099	6.092567
Diphenyl ether	Benzene and substituted derivatives	1.190736	2.148731	1.103485
L-Cystathionine	Amino acid metabolomics	2.034695	7.419325	2.891288
3-Hydroxyhippuric acid	Organic acid and its derivatives	1.030264	6.878891	2.782176
Vanillic acid	Organic acid and its derivatives	1.674148	3.891708	1.960404
Methylmalonic acid	Organic acid and its derivatives	1.391963	2.752149	1.460559
Aminomalonic acid	Organic acid and its derivatives	1.477349	2.632414	1.396387
Glutaric acid	Organic acid and its derivatives	2.310351	2.204869	1.140693
3,4,5-Trimethoxycinnamic acid	Organic acid and its derivatives	1.179124	2.199951	1.137471
Methylparaben	Benzoic acid and its derivatives	1.033092	6.023043	2.590493
γ-glutamyl-leucine	Amino acid metabolomics	1.750045	2.192277	1.13243
Hexanoyl glycine	Amino acid metabolomics	1.785677	3.271286	1.709858
N-Isovaleroylglycine	Amino acid metabolomics	1.558525	3.061615	1.614293
Succinic acid	Amino acid metabolomics	1.496548	2.774461	1.472208

## Data Availability

Data is contained within the article.

## Supplementary Materials

The following supporting information can be downloaded at: https://www.mdpi.com/article/10.3390/foods12030560/s1, Figure S1: The goats would like to ingest *A. sinensis* leaves; Figure S2: An orthogonal partial least squares-discriminant analysis (OPLS-DA) score plot of serum and muscle metabolome distribution according to the diet of goats; Table S1: Bioactive compounds identified from *A. sinensis* leaves; Table S2: UPLC-MS/MS metabolomics analysis of serum samples from goats fed *A. sinensis* leaves; Table S3: UPLC-MS/MS metabolomics analysis of muscle samples from goats fed *A. sinensis* leaves; Table S4. The supported information of PCA scores plot of serum metabolome distribution according to the diet of goats; Table S5. The supported information of PCA scores plot of muscle metabolome distribution according to the diet of goats.
