# Supplementary material for: Feeding Aquilaria sinensis Leaves Modulates Lipid Metabolism and Improves the Meat Quality of Goats
Source: Foods. 2023 Jan 27;12(3):560. doi: 10.3390/foods12030560 (PMC9914005; doi:10.3390/foods12030560)
Supplement: Supplementary file 1 [file foods-12-00560-s001.zip › Figure S2.pdf]

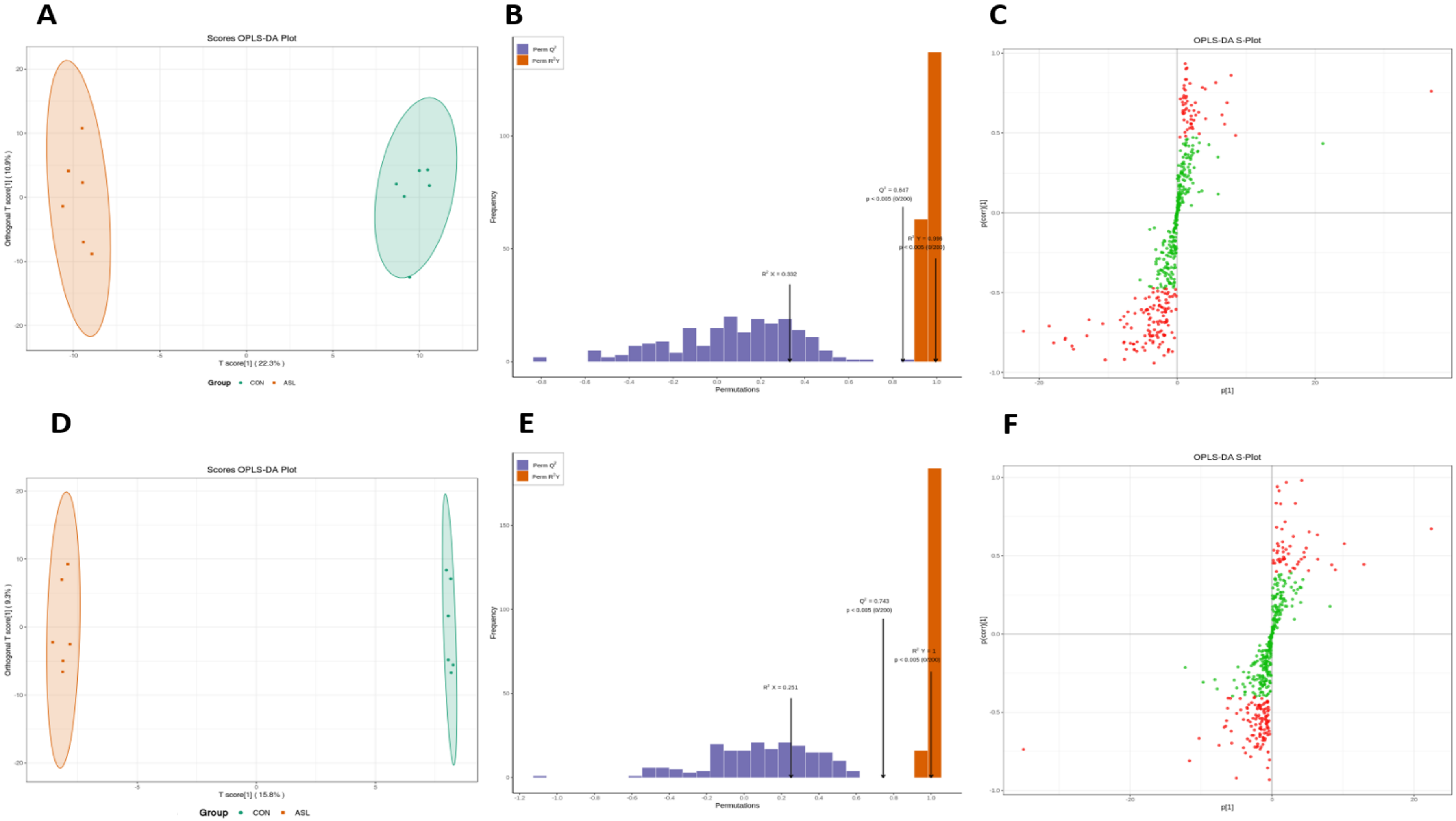

Figure S2. OPLS-DA scores plot of serum and muscle metabolome distribution according to the diet of goats.

(A) OPLS-DA scores plot of serum metabolome distribution according to the diet of goats. (B) Permutation tests for OPLS-DA model of serum metabolome. (C) S-plot for OPLS-DA model of serum metabolome. (D) OPLS-DA scores plot of muscle metabolome distribution according to the diet of goats. (E) Permutation tests for OPLS-DA model of muscle metabolome. (F) S-plot for OPLS-DA model of muscle metabolome. CON: a normal diet, ASL: a diet containing 20% *A. sinensis* leaves. Red indicated VIP > 1, while green indicated VIP ≤1.
