# Supplementary figures and images for: Feeding Aquilaria sinensis Leaves Modulates Lipid Metabolism and Improves the Meat Quality of Goats
Source: Foods. 2023 Jan 27;12(3):560. doi: 10.3390/foods12030560 (PMC9914005; doi:10.3390/foods12030560)

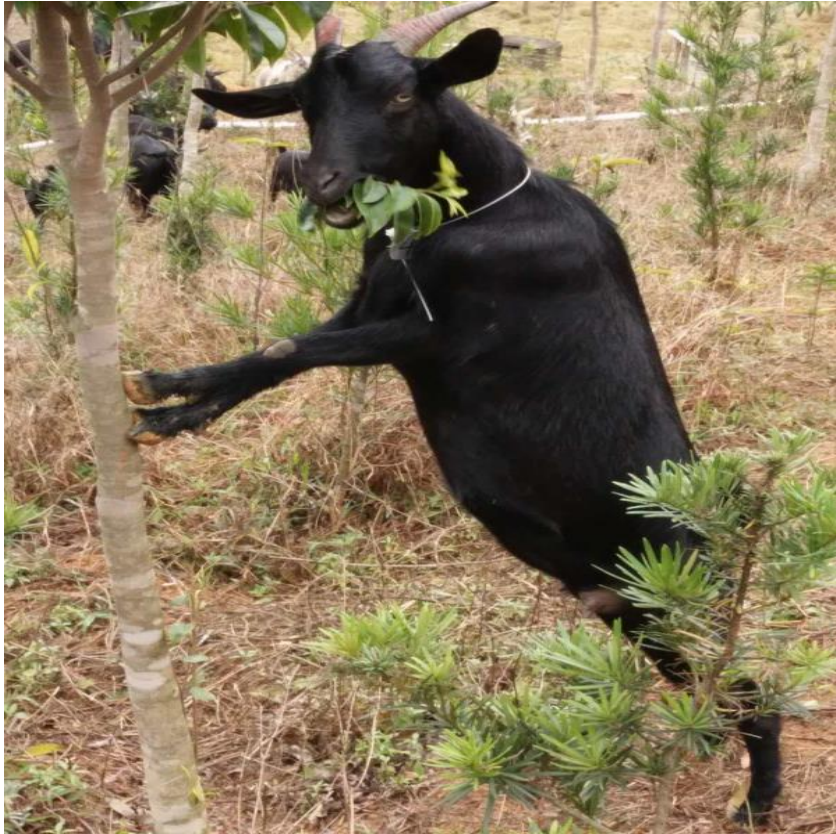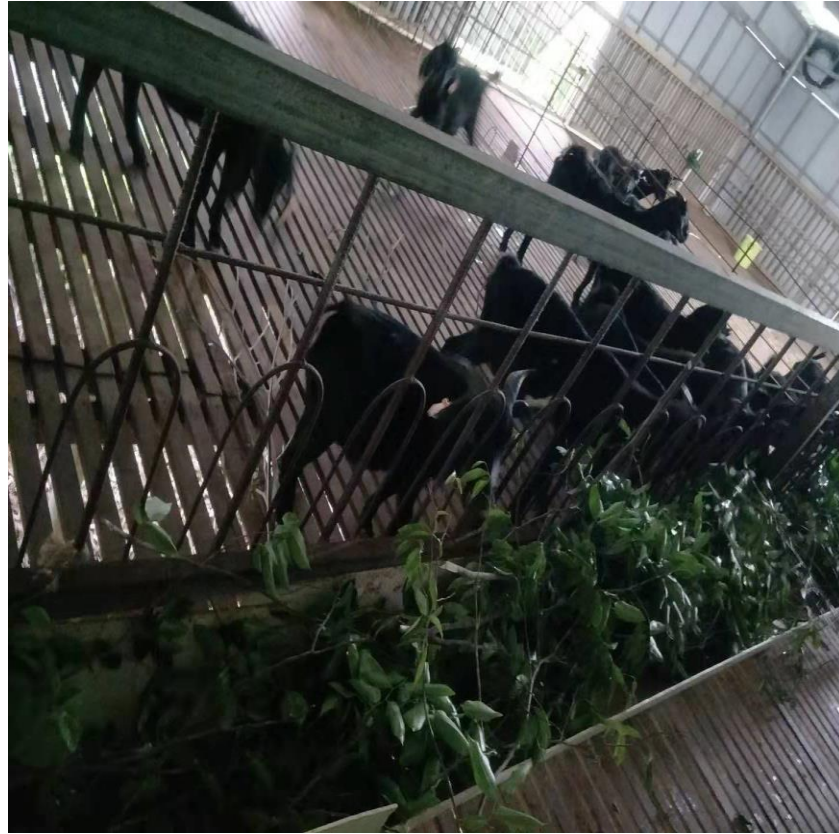

Figure S1. The goats would like to ingest *Aquilaria sinensis* leaves.

Supplement: Supplementary file 1 [file foods-12-00560-s001.zip › Figure S1.pdf]
